# Mood Disorders, Accelerated Aging, and Inflammation: Is the Link Hidden in Telomeres?

**DOI:** 10.3390/cells8010052

**Published:** 2019-01-15

**Authors:** Alessio Squassina, Claudia Pisanu, Roberta Vanni

**Affiliations:** 1 Department of Biomedical Sciences, Section of Neuroscience and Clinical Pharmacology, University of Cagliari, 09042 Monserrato Cagliari, Italy; squassina@unica.it; 2 Department of Psychiatry, Dalhousie University, Halifax, NS B3H 2E2, Canada; 3 Department of Neuroscience, Unit of Functional Pharmacology, Uppsala University, 752 39 Uppsala, Sweden; 4 Department of Biomedical Sciences, Unit of Biology and Genetics, University of Cagliari, 09042 Monserrato Cagliari, Italy

**Keywords:** telomere shortening, inflammation, aging, bipolar disorder, major depression, mood disorders, lithium, antidepressants, mood stabilizers

## Abstract

Mood disorders are associated with an increased risk of aging-related diseases, which greatly contribute to the excess morbidity and mortality observed in affected individuals. Clinical and molecular findings also suggest that mood disorders might be characterized by a permanent state of low-grade inflammation. At the cellular level, aging translates into telomeres shortening. Intriguingly, inflammation and telomere shortening show a bidirectional association: a pro-inflammatory state seems to contribute to aging and telomere dysfunction, and telomere attrition is able to induce low-grade inflammation. Several independent studies have reported shorter telomere length and increased levels of circulating inflammatory cytokines in mood disorders, suggesting a complex interplay between altered inflammatory–immune responses and telomere dynamics in the etiopathogenesis of these disorders. In this review, we critically discuss studies investigating the role of telomere attrition and inflammation in the pathogenesis and course of mood disorders, and in pharmacological treatments with psychotropic medications.

## 1. Introduction

**Figure 1:**
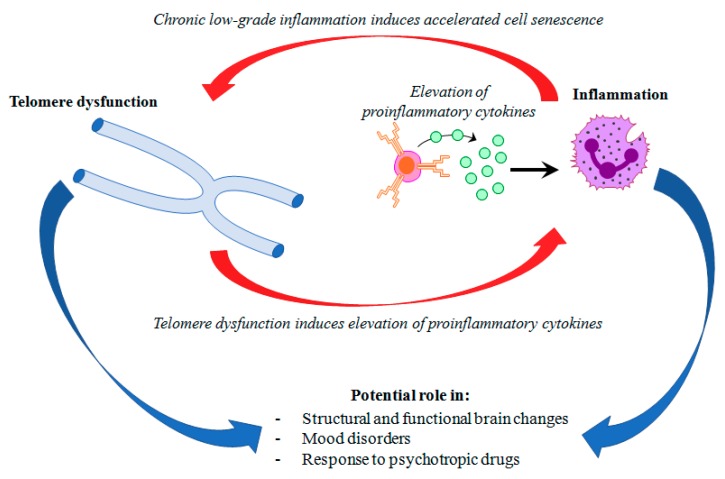
Hypothetical interplay between telomere dysfunction and low-grade inflammation in mood disorders.

Mood disorders affect more than 400 million people world-wide [1]. Major depressive disorder (MDD) and bipolar disorder (BD) represent the most common mood disorders, and are associated with a high level of disability, and a substantial reduction in life expectancy, compared to the general population [2]. A recent systematic review and meta-analysis published by Walker and colleagues [3] showed that almost 70% of deaths among people with psychiatric disorders are due to natural causes. Most of these deaths are accounted for by chronic physical medical conditions that are usually associated with aging, such as cardiovascular, respiratory, and infectious diseases, diabetes, and hypertension [3,4]. These disorders are also characterized by a persistent inflammatory state, a feature that is shared with mental illness. This evidence has led to the hypothesis that accelerated aging and inflammation may play a central role in the etiopathogenesis of psychiatric disorders [5] (Figure 1). 

Most of the studies exploring telomere dynamics and inflammatory markers in mood disorders have been so far carried out in peripheral blood. Shorter leukocyte telomere length (LTL), as well as increased levels of circulating pro-inflammatory cytokines, have been reported in mood disorders [6,7,8]. Telomere shortening (TS) is a hallmark of cellular aging, and while telomeres shorten physiologically with each cell division, this shortening rate can be increased by allostatic load and inflammatory insults [9], thus affecting the aging process. 

On one hand, most of the studies published so far reported shorter telomeres in mood disorder patients compared to healthy individuals [10]; on the other hand, the investigation on the potential interaction between the inflammatory processes and telomere shortening in the etiology and progression of mood disorders has been scarce. While the hypothesis of the involvement of a “telomere-inflammation network” in mood disorders is supported by data from independent investigations, an important issue has yet to be elucidated: do altered telomere–inflammation dynamics represent a manifestation of the disease progression (and therefore of the allostatic load that is also contributed by the disorder) and/or of the impaired quality of life of affected patients, or do they represent causative factors of the disorder, and as such, are a genetically-determined component? We can anticipate that the available literature does not comprehensively answer the question, mainly as a consequence of its complexity. The few published studies implementing a longitudinal design seem to suggest that inflammation and TL influence each other in a bidirectional way, modulating mood symptoms and the susceptibility of individuals to mood disorders and comorbid medical conditions that are associated with aging. 

In this article, we critically review the existing literature investigating the role of telomere attrition in the pathogenesis and course of mood disorders. We provide a brief overview on telomere biology and the role of inflammation in telomere dynamics and mood disorders. A special focus is given to studies integrating the contribution of important variables that have been suggested to affect telomere length (TL) such as age, sex, body mass index (BMI), and smoking, as well as studies investigating the association between TL, inflammation, and clinical characteristics, such as the duration and stage of illness, symptom severity, and suicidal behavior. Considering their potential impact on the field, we specifically discuss prospective studies on mood disorder patients. Findings on the role of medications, as well as the complex interplay between telomeres and inflammation in mood disorders, are also discussed in detail.

## 2. Telomeres 

The ends of chromosomes are specialized structures [11] that, in humans, primarily consist of highly-conserved nucleotide-repeat regions containing 5′-TTAGGG-3′ DNA tandem repeats (telomere) and telomere-associated shelterin proteins [12]. This specialized end is crucial for the integrity of the genome, distinguishing the natural termination of the chromosome from possible terminations, due to double-strand DNA breaks, and protecting chromosomes from end-to-end fusions, misrepair, and degradation. In adult proliferating tissues, the telomeres undergo progressive shortening at each cell cycle, until the cells reach sufficiently short telomeres to undergo growth arrest and enter into a senescent status, thus remaining viable, but not dividing [13]. This progressive shortening is mainly counteracted by the enzyme telomerase, an RNA-dependent DNA polymerase that can add telomeric repeat sequences at the ends of chromosomes [11]. However, telomerase is absent in the vast majority of somatic differentiated cells, being active only in a reduced number of adult normal cell types, namely in spermatogonia and in subsets (normal transit-amplifying cells) of proliferating somatic adult progenitor cells [14,15]. Telomere maintenance is therefore crucial in cell functioning, and as such, it is a dynamic, finely regulated process: on one hand, pro-apoptotic processes can be activated when telomeres reach a critical shortening threshold; on the other hand, the inhibition of telomere shortening may trigger the transition of normal cells to cancer cells [16]. 

The physiological shortening of telomeres represents a biological marker of cellular aging. However, it is now well understood that the degree of telomere shortening can be accelerated by several stimuli and insults, including oxidative stress, inflammation, and hormones, as well as genetic variants in genes involved in telomere biology [17,18]. Chronic and age-related medical conditions, including mood disorders, have been associated with telomere shortening [16]. Interestingly, several of the factors inducing accelerated telomere shortening, such as inflammation and oxidative stress, have also been implicated in mood disorders, supporting the hypothesis of a complex interplay between inflammation and telomere shortening in mood disorders. 

### 2.1. Telomeres and Inflammation

There is extensive evidence supporting an interaction between inflammatory and telomere pathways in human disorders. Several chronic inflammatory diseases have been associated with telomere shortening, including cardiovascular and metabolic disorders, substance abuse, autoimmune, and infectious diseases [19]. While the complexity of the crosstalk between the immune and inflammatory response and telomere maintenance has yet to be elucidated, both in vitro and in vivo evidence shows that chronic inflammation interacts with TL in a bidirectional way. Chronic low-grade inflammation induced by knockout of the gene encoding for a subunit of the transcription factor nuclear factor kappa-light-chain-enhancer of activated B cells (NF-κB) was shown to induce accelerated cell senescence and telomere dysfunction in mice, in the absence of external stimuli [20]. On the other hand, in telomerase-deficient mice, telomere dysfunction was shown to induce the elevation of different proinflammatory cytokines in the absence of external stimuli, and in proportion to the extent of telomere shortening [21,22]. The main mechanism explaining the telomere dysfunction induced by chronic inflammation seems to be oxidative stress, i.e., an imbalance between the production of reactive oxygen species and the cellular systems deputed to neutralize them [23]. In this sense, it is important to note that telomeres are particularly sensitive to oxidative damage, and that they have a less efficient DNA damage response [24]. Very recently, Criscuolo and coworkers [25] investigated whether mechanisms involved in telomere attrition may be revealed by a proteomic approach. They studied TL in relation to the proteomic profile changes after the induction of an acute inflammatory immune response in mouse splenocytes. They reported shorter telomeres in older rats, and demonstrated an age-dependent change of the proteome. The finding of shorter telomeres in old mouse could be explained by a transient inhibition of telomerase activity, owing to the trade-off between telomere maintenance and the immune response. 

Another mechanism possibly explaining the relationship between inflammation and telomere dysfunction might involve alterations of the integrity of the blood–brain barrier (BBB). Telomerase-deficient mice show larger infarct volumes and worse neurologic deficits, compared to mice with normal telomerase function, after permanent middle cerebral artery occlusion [26]. This effect might be explained by a higher increase in superoxide levels, as well as reduced glutathione antioxidant protection in telomerase-deficient mice. In this study, telomerase reverse transcriptase (TERT) deficiency was also associated with the elevation of inflammatory cytokines, as well as a decreased expression of proteins playing a crucial role in the regulation of BBB integrity [26]. 

In humans, cumulative inflammatory loads reflected by high levels of inflammation markers such as C-reactive protein (CRP) [27,28], fibrinogen [27], interleukin 6 (IL-6) [28,29,30], and tumor necrosis factor (TNF)-a [30] have been cross-sectionally associated with shorter TL, by a number of studies. Lin and coworkers [31] extended this evidence in a prospective study on a sample of 20 healthy mothers of children with autism (caregivers) and 19 mothers of neurotypical children (controls). Peripheral blood mononuclear cells (PBMC) from the included subjects were stimulated in vitro with phytohemagglutinin (PHA). The stimulation increased the expression levels of genes included in proinflammatory pathways only in cells from caregivers, suggesting an exaggerated proinflammatory reactivity response. In the whole sample, higher expression levels of proinflammatory genes (including NF-kB, interleukin 1 beta (IL-1b), IL-6, interleukin 6 receptor (IL-6R), interleukin 8 (IL-8), interleukin 17C (IL-17C), Erk1, CAMP-responsive element-binding protein 1 (CREB1) and phosphatidylinositol-4,5-bisphosphate 3-kinase catalytic subunit alpha (PIK3CA)) were associated with shorter TL 15 months later [31]. As a whole, these results show that stress-induced chronic inflammation reduces TL in a relatively short period of time. 

On the other hand, another recent study conducted in the Baltimore Longitudinal Study on Aging cohort found no significant correlation between the rate of change in TL, and levels of the inflammatory cytokines interferon gamma, IL-6, and interleukin 10 (IL-10) in a sample of 465 individuals aged from 21 to 88 years at first visit, with an average duration of follow-up equal to 13 years [32]. 

Overall, these studies suggest that the relationship between TL and inflammatory cytokines is complex, and might be further modulated by other variables, such as mood disorders and chronic stressors. Studies exploring the relationship between TL and inflammatory cytokine levels in mood disorders will be discussed in detail in Section 5. 

### 2.2. Inflammation and Mood Disorders

The role of dysfunctions in immune–inflammatory pathways in the pathophysiology of mood disorders is supported by a large number of studies [33,34]. Overall, the levels of proinflammatory cytokines have been reported to be higher in BD patients compared to other mood disorders or healthy controls in several studies. A systematic review and meta-analysis including 13 studies (comprising 556 BD patients and 767 healthy controls, and evaluating 15 different cytokines) showed that levels of TNF-α, the soluble tumor necrosis factor receptor type 1 (sTNF-R1) and the soluble inlerleukin-2 receptor (sIL-2R), were elevated in manic patients compared with healthy control subjects, while levels of sTNF-R1 and TNF-a were elevated in manic patients, compared to euthymic patients [35]. 

Interestingly, levels of endogenous cytokines have been shown to correlate with the volumes of different brain structures. In a recent paper by Chen and colleagues [36], several brain regions (the orbitofrontal cortex, lingual gyrus, inferior frontal cortex, middle frontal cortex, and planum polare) were significantly smaller in patients with BD, than in those with MDD. These gray matter volume differences were negatively correlated with soluble IL-6 receptor levels, suggesting high levels of this cytokine negatively impact on brains functions and structure. 

Indeed, low-grade inflammation might induce structural and functional changes on the brain via different mechanisms [37]. For instance, cytokines may either pass through leaky regions in the BBB, or bind to peripheral afferent nerve fibers such as the vagus nerve, thus stimulating ascending catecholaminergic fibers in the brain [34]. Importantly, inflammatory cytokines can impair synthesis, as well as the synaptic availability of monoamines [38,39]. Consistent with these observations, a recent large meta-analysis of genome-wide association datasets implicated genes involved in cytokine and immune responses in the pathogenesis of MDD [40]. Low-grade inflammation has also been suggested to modulate the response to antidepressants [41,42,43,44].

Moreover, antidepressant treatment decreases the peripheral levels of inflammatory cytokines, including IL-6, IL-10, and TNF-α, as shown by recent meta-analyses [37,45]. 

Altered cytokine levels appear to be a transdiagnostic marker in psychiatry. In a recent study, Goldsmith and coworkers [46] performed a meta-analysis of blood cytokines in acutely and chronically ill patients with schizophrenia, BD, and MDD. Overall, the patterns of cytokine alterations (IL-6, TNF-α, sIL-2R, and IL-1RA) showed a certain degree of similarities among the three diagnostic groups during the acute and chronic phases of illness. These findings suggest a possible common contribution of altered inflammatory and immune responses in psychiatric disorders.

## 3. Telomeres and Mood Disorders

### 3.1. Telomeres and Bipolar Disorder

The majority of studies published so far explored peripheral TL in BD, using genomic DNA extracted from leukocytes [47,48,49,50], peripheral blood mononuclear cell (PBMC) [51,52] or buccal smears [53]. Overall, findings suggest that BD patients have a significant decrease of peripheral TL, compared with healthy controls [47,51,52,54]. This effect, also confirmed by a recent meta-analysis [55], seems to be independent from mood state, as shorter telomeres have been reported in BD patients in euthymic, depressed, and manic states [55]. Shorter TL has also been associated with familial risk for BD [53]. 

To date, only one study reported increased TL in patients with BD, compared with healthy individuals [48]. However, in the study by Martinsson and coworkers, the large majority of patients were chronically treated with lithium at the time of recruitment. Several studies suggest that lithium may counteract telomere shortening, and as such, the discrepancy in findings might be explained by the effect of chronic exposure to lithium. The correlation between pharmacological treatments and TL will be further discussed in Section 4.1 of this review. 

Several studies have also explored the correlation between TL and the clinical features of BD. Two recent studies compared early- or late-stage BD patients. The study by Kose Cinar and colleagues [54], showed shorter LTL in late-stage BD patients compared to early-stage and healthy controls (*p* < 0.001). Early-stage patients were defined as those with less than five episodes, while late-stage were those with more than 10 episodes. In another study, Barbè-Tuana et al. [56] measured LTL in BD patients where the disease stage was characterized as I and IV, based on Kapczinski’s criteria [57]. Findings showed that telomere shortening was already present in patients in the early stages of disease, compared to the controls, and reported no significant difference in LTL between early vs late stage. The meta-analysis of these two studies conducted by Huang et al. [55] showed that although both groups of patients had significantly shorter LTL compared to the controls, late-stage patients had a further reduction of LTL compared to early-stage patients. However, these results should be carefully interpreted, due to the different methods used by the two studies to define the stage of disease, as well as due to the limited sample size.

Other clinical variables, such as the duration of illness [51,54] and the number of lifetime hypomanic or manic episodes [47,48,51,53,54] have not been found to correlate with TL in BD. However, contrasting findings have been reported regarding the number of depressive episodes. Some studies reported a negative correlation between LTL and number of depressive episodes [48,51], while others found no association [47,50,53,54]. Furthermore, no study reported an association between LTL and the number of suicide attempts [47,50,54].

To the best of our knowledge, the only study investigating the relationship between BD and genetic variants in genes involved in telomere biology was carried out by Wei and coworkers [58]. In this study, the minor allele of the human TERT (hTERT) polymorphism rs2736100 was associated with the number of depressive episodes in patients with BD type 1 who were good responders to lithium, but not in non-responders [58]. On the other hand, polygenic risk scores for BD, MDD, or schizophrenia were not associated with peripheral TL in a cohort of healthy individuals [59]. Further studies will be needed to understand if cumulative evaluations of the genetic risk for psychiatric disorders may be of help in identifying patients who are at a higher risk of accelerated aging.

While the large majority of published studies measured peripheral TL, few studies explored the putative association between BD and shorter TL in postmortem brains. However, findings from these studies so far do not support an association between BD and TL in cerebellar gray matter [60], or in different brain regions, including the dorsolateral prefrontal cortex, hippocampus, amygdala, nucleus accumbens, and substantia nigra [61]. Overall, the limited number of postmortem data on TL greatly limits our interpretation of the putative correlation between peripheral and brain TL in BD.

Interestingly, a recent study by Powell and colleagues [53] suggested that peripheral TL might predict brain volume. In this study, TL measured on buccal DNA explained a substantial variance in hippocampal volume measured with magnetic resonance in a sample of bipolar patients, first-degree relatives, and unrelated healthy controls. This result supports the hypothesis that TL might represent a marker of hippocampal vulnerability, as previously suggested [62]. A subsequent study further explored the association between peripheral TL and functional brain activation and connectivity, in a sample comprising patients with BD and first-degree relatives, as well as healthy volunteers [63]. TL was positively associated with increased face-related activation in the amygdala, during a task in which participants were asked to identify facial emotions. This association was observed, regardless of the diagnosis status. Furthermore, a polygenic risk score for TL was positively associated with medial prefrontal cortex activation [63]. These results support the existence of a link between TL and emotional brain activity.

### 3.2. Telomeres and Major Depressive Disorder

As in the case of BD, the majority of studies reported shorter TL in MDD. The most recent meta-analysis pooling results from 38 studies found that depression, as well as depression severity, were significantly associated with shorter TL (*p* < 0.00001 and *p* = 0.03, respectively) [64]. Furthermore, Vance and coworkers [65] conducted a prospective longitudinal study to evaluate the association between depression at baseline, and a change in LTL over two years [65]. A diagnosis of MDD was found to prospectively predict LTL shortening after correcting for age, sex, and BMI, in a sample of 67 well-characterized MDD patients, and 50 healthy controls [65]. However, LTL was not associated with a change of MDD symptoms severity or duration during the follow-up. A larger study published by Révész and coworkers in 2016, including 2750 participants of the Netherlands Study of Depression and Anxiety, showed that the relationship between psychopathology and LTL was modulated by levels of inflammatory markers (CRP and IL-6) as well as by waist circumference, triglycerides, high-density lipoprotein cholesterol, and cigarette smoking [66]. Remarkably, this study provides significant support for the hypothesis of an interaction between factors involved in inflammation and telomere dynamics in mood disorders. 

Few studies have investigated the role of genetic variants located in genes that are relevant for telomere biology. Wei and coworkers [58] showed an association between the intronic hTERT rs2736100 single nucleotide polymorphism (SNP) and the diagnosis of depression among subjects with no experience of childhood adversity. Michalek and coworkers [67] used a different approach and applied Mendelian randomization to investigate the association of TL and risk for recurrent MDD in a large UK sample, including 1628 MDD cases and 1140 controls. The authors found that the T allele of rs10936599, which is located upstream of the human telomerase RNA component (hTERC) gene, and which is associated with shorter LTL, increased the risk for childhood-onset MDD relative to controls or to adult-onset MDD. These results suggest that the genetic risk for shortened LTL is able to increase the risk for MDD, and specifically for childhood-onset MDD [67]. Conversely, a recent study did not find any association between a polygenic risk score, including nine SNPs that were previously associated with inter-individual variation in TL [17,18,68] and the risk of lifetime depression in a large sample of 17,693 female participants of European ancestry from the Nurses' Health Study (NHS) [69]. Potential explanations of the discrepancies observed among the studies might be found in the different definitions used to define MDD, as well as in our still-limited knowledge of genetic variants that are able to explain inter-individual variability in TL. 

As in the case of BD, few studies have investigated the interplay between peripheral TL and either TL in brain or brain volume. A recent study conducted by Wolkowitz and coworkers [62] found a positive association between hippocampal volume and PBMC telomerase activity, but not TL, in a sample including 25 un-medicated patients with MDD [62]. In a postmortem brain study, Mamdani and coworkers [61] showed a significant telomere shortening in the hippocampus of MDD subjects compared with controls [61]. Importantly, single-cell populations in the brain might be differentially vulnerable to telomere shortening, as suggested by a study showing reduced TL in oligodendrocytes, but in not astrocytes derived from post-mortem prefrontal cortex brain samples from patients with MDD, compared to healthy controls [70]. Thus, future investigations on telomere shortening in the brain should consider accounting for the effects of different cell types in their experiments. 

## 4. Telomeres and Psychotropic Medications 

### 4.1. Lithium and Telomeres

Robust preclinical and clinical evidence suggests that lithium may exert a counteractive effect on telomere shortening. Using a preclinical model of telomere dysfunction, Wei and coworkers [71] showed that a six-week lithium treatment was able to normalize *TERT* expression and telomerase activity in the hippocampus of Flinders Sensitive Line mice, possibly via the upregulation of β-catenin [71]. In this study, normalization of the telomerase gene expression and protein activity was not accompanied by increase of TL, suggesting that changes in TL might occur in a much slower way, compared to changes in the enzyme activity [71]. Intriguingly, Cardillo and coworkers [72] showed that chronic lithium treatment (eight months) increased TL in the hippocampus and parietal cortex in a transgenic mouse model of Alzheimer’s disease [72]. Consistent with these observations, clinical studies suggest that the duration of lithium treatment is positively associated with TL in BD patients with a history of long-term treatment with this drug [48,50]. Two recent studies showed longer telomeres in BD patients on lithium by using two different approaches: the study by Powell and colleagues reported longer TL measured on buccal DNA in BD patients treated with lithium, compared with patients not on lithium [53], while the study by Coutts and colleagues reported longer TL on whole blood DNA from patients with long-term lithium treatment, compared with short-term lithium users [73]. The latter study also showed that a polygenic risk score for TL explained a higher proportion of the variance in TL in patients with long-term lithium treatment, compared to short-term users or lithium-naïve patients [73]. These findings support the need to further investigate the putative effect of lithium on genes playing a role in the regulation of TL.

To date, two studies showed a correlation between the clinical response to lithium treatment, and TL. Martinsson and coworkers showed longer LTL in BD patients responding to lithium, compared to non-responders [48], while Kose Cinar et al., [54] showed longer LTL in remitted BD patients after treatment with lithium plus antipsychotics. On the other hand, another study showed no association between LTL and lithium response in BD [50]. The paucity and discrepancy of available data support the need for further investigation into the role of TL in modulating the clinical efficacy of lithium treatment in BD. Although the mechanisms through which lithium might counteract telomere shortening are not clear, a putative effect of lithium on telomerase expression and activity has been suggested. Specifically, in vitro lithium treatment was shown to increase hTERT expression in human-derived neural progenitor cells (NPCs), although the effect was not statistically significant, possibly due to the low number of samples included [50]. As further discussed in Section 4.2, the role of telomerase in modulating the clinical efficacy of psychotropic drugs is yet to be elucidated, as studies have so far reported contrasting findings. 

### 4.2. Antidepressants and Telomeres 

Few studies investigated the role of antidepressants in modulating TL. In mice, telomerase dysregulation induced by chronic mild stress in the hippocampus, which was associated with depression-like behaviors and reduced hippocampal neurogenesis, was rescued by treatment with the antidepressant fluoxetine [74]. In humans, telomerase activity was found to be elevated in PBMCs from subjects with MDD, compared with healthy controls, as well as being correlated with MDD severity [75]. The same study showed that lower pre-treatment telomerase activity, as well as a greater increase in telomerase activity during treatment, predicted the response to sertraline [75]. Another study suggested that shorter TL might represent a marker of poor response to selective serotonin reuptake inhibitors (SSRI). In this study, including 27 drug-free patients with MDD, a shorter TL at baseline was associated with less improvement in the negative but not positive affect scale in patients treated with SSRI antidepressants for eight weeks [76]. Moreover, longer LTL was also associated with better antidepressant responses to the adjuvant PPAR-γ agonist pioglitazone, in a sample including 42 patients with unremitted depression [77]. A recent study found antidepressant use to be associated with shorter TL and a higher prevalence of aging-related diseases independently from depression diagnosis [59]. Overall, studies on the effect of lithium and antidepressants suggest that telomerase might be modulated by psychotropic medications, but the direction of the induced changes and the correlation of this effect with clinical efficacy needs further investigation. 

## 5. Mood Disorders, Telomeres, and Inflammation

The link between mood disorders, telomeres, and inflammation is supported by a number of independent investigations. One of the first studies to investigate this link in MDD was conducted by Wolkowitz and coworkers [78]. In this study, including 18 un-medicated MDD subjects and 17 controls, TL was correlated with levels of IL-6 and F2-isoprostane/vitamin C ratio, which represent two markers of inflammation and oxidative stress, respectively [78]. Levels of IL-6 were inversely correlated with TL in subjects with MDD, while a higher isoprostane/vitamin C ratio was associated with reduced TL in both groups. Only subjects with a more chronic course of MDD differed from controls in terms of LTL [78], suggesting that accelerated telomere shortening might represent a consequence of a longer exposure to depression, rather than a predisposing factor [78]. In a recent study, Crawford and coworkers [79] showed that a self-reported history of depression and of an inflammatory disorder significantly interact in predicting whole blood DNA methylation at specific differentially methylated regions, such as the one located in the leukotriene B-4 receptor (LTB4R2) gene [79]. Moreover, the authors used weighted gene co-methylation network analysis (WGCNA) to identify networks of co-methylated modules that are associated with depression, TL, and IL-6 levels. This analysis identified a gene co-methylation module (the “Turquoise module”) associated with the interaction between history of depression and inflammatory disorders, as well as with TL and levels of IL-6 [79]. The gene ontology enrichment analysis showed that this module was enriched for pathways related to immune function. This study supports the hypothesis that epigenetic mechanisms, such as DNA methylation, might play an important role in mediating the interaction between depression, inflammation, and TL. 

Another study conducted in post-mortem brain samples from the prefrontal cortex (BA10) found shorter TLs in oligodendrocytes but not astrocytes derived from patients with MDD, compared to healthy controls [70]. This observation was accompanied with reduced expression levels of the gene encoding the telomerase enzyme, as well as of genes encoding important oxidative defense enzymes, such as superoxide dismutase (SOD1 and SOD2), catalase (CAT), and glutathione peroxidase (GPX1) [70].

In a study by Osler and coworkers [80], the authors explored the interplay between stressful life events, clinical features (including the characterization of depressive symptoms) and measures of inflammation and biological stress in a large cohort of Danish men. Levels of TL, IL-6, IL 10, and CRP were available for a sample of 324 men. Findings showed that early stressful events were associated with shorter telomeres in middle-aged men, and that the largest proportion of this association was mediated through depressive mood and CRP.

Fewer studies have investigated the link between telomeres and inflammation in patients with BD. A recent study including 36 patients with BD type 1, 39 siblings, and 44 healthy controls, showed that patients had shorter telomeres, as well as increased levels of the proinflammatory cytokines IL-6 and IL-10, compared to healthy controls, but to not siblings [81]. Importantly, the patients also showed low glutathione peroxidase activity compared to the controls, supporting the putative role of oxidative stress in the interplay between mood disorders, TL, and inflammation.

## 6. Longitudinal Studies Exploring Telomere Length and Inflammation in Mood Disorders 

As we pointed out in the Introduction section, it is not yet clear to what extent the telomere shortening observed in mood disorders is a consequence of telomere attrition (also attributable to the disorder) and/or a causative, predisposing factor in susceptible individuals. In this section, we discuss in more detail longitudinal studies that are already considered in this review, but with a focus on the insights that could be provided by the longitudinal approach. 

Only a few studies implemented a prospective-longitudinal design to explore the correlation between disease progression (or disease symptoms and clinical variables), TL and inflammation. The study by Kose Cinar [54] showed that telomeres were shorter in drug-naïve BD patients, compared to controls, but that eight weeks of lithium treatment increased LTL. Moreover, LTL was shorter in late-stage BD patients (higher numbers of episodes) compared to early-stage and controls. While these findings do not allow for the elucidation of the causative role of telomere shortening, they suggest that disease severity and progression represent important factors contributing to telomere attrition, and that treatment with lithium increases TL, as previously shown in other studies. Although not all of the findings on the correlation between the clinical efficacy of lithium and TL are concordant with the Kose Cinar paper, it is important to note that most of the previous investigations used a retrospective design. 

The study by Wolkowitz and coworkers [75] provided further support for the longitudinal effects of psychotropic medications on telomere dynamics. Indeed, findings showed that telomerase activity was increased by the antidepressant sertraline after eight weeks of treatment in patients with relatively lower pretreatment telomerase activity, and that this increase was larger in patients who responded better to the treatment. 

The study by Vance and coworkers [65] showed that the diagnosis of MDD could prospectively predict LTL shortening after two years, though LTL was not different at baseline between MDD and the controls. Again, this study suggests that major depressive disorder could contribute to telomere shortening, rather than shorter telomeres predisposing individuals to mood disorders. 

To the best of our knowledge, only one study has so far explored the correlation between early-life exposure to stressors, mood symptoms, telomeres, and inflammation. The study by Osler et al. [80] showed that childhood stressful events were associated with shorter TL in middle-aged men, and that 30% of this effect was accounted for by depressive mood and CRP levels. 

While these findings strongly support the interplay between inflammation, telomere shortening, and mood symptoms, the lack of data on the longitudinal measurement of TL and inflammatory markers do not allow us to understand the longitudinal trajectory of altered telomere and inflammation dynamics in mood disorders. 

## 7. Discussion 

The role of the interplay between inflammation and telomeres in mood disorders has been explored in a large number of independent studies, but the interaction between altered telomeres and inflammation dynamics has been rarely explored in the same experimental setting. Elevated levels of inflammatory markers have been reported in mood disorders, supporting the hypothesis that inflammation could play a causal role in these disorders [82]. However, while published data are more convergent and robust in supporting the hypothesis of the causative role of inflammation, the same cannot be implied for telomeres. Findings suggest that mood symptoms and disease severity might significantly contribute to telomere attrition, but it is not clear whether pre-existing shorter telomeres contribute to disease onset, or if the onset of the disease contributed to accelerating the physiological shortening of “normal” telomeres. This issue has been hard to overcome, as ideal studies on the trajectory of altered telomere and inflammation dynamics should involve at-risk individuals (e.g., individuals not presenting the disease, but with a risk of developing it in the future, such as in the offspring of affected individuals) and follow them for decades. The inclusion of clinical variables that are potentially important for exploring the correlation between accelerated aging, mood disorders, and telomere shortening, such as comorbidity and age-related conditions, has been overseen in most of the studies published so far. Future investigations should explore the role of telomere shortening in the reported higher incidence of comorbid-age related conditions in mood-disorder patients compared to the general population. 

## 8. Conclusions

Overall, the available literature suggests that inflammation significantly contributes to telomere attrition, and that mood disorder patients are more vulnerable to low-grade inflammation and shorter telomeres, compared to healthy individuals. However, the lack of prospective studies does not allow for the support or exclusion of a causative role of altered inflammation and telomere dynamics in these disorders. The effect of psychotropic medications on telomere dynamics has been reported by several studies, and it appears to be particularly robust for lithium. It seems that this effect could be at least partly mediated through the modulation of telomerase activity and hTERT expression, even though data are still scarce and not concordant in this respect. On the other hand, the correlation between this modulatory effect by psychotropic medications and their clinical efficacy is less clear, and requires further investigation.
